# Finding gene network topologies for given biological function with recurrent neural network

**DOI:** 10.1038/s41467-021-23420-5

**Published:** 2021-05-25

**Authors:** Jingxiang Shen, Feng Liu, Yuhai Tu, Chao Tang

**Affiliations:** 1 Center for Quantitative Biology, Peking University, Beijing, China; 2 School of Physics, Peking University, Beijing, China; 3 IBM T. J. Watson Research Center, Yorktown Heights, New York, USA; 4 Peking-Tsinghua Center for Life Sciences, Peking University, Beijing, China

**Keywords:** Regulatory networks, Reverse engineering

## Abstract

Searching for possible biochemical networks that perform a certain function is a challenge in systems biology. For simple functions and small networks, this can be achieved through an exhaustive search of the network topology space. However, it is difficult to scale this approach up to larger networks and more complex functions. Here we tackle this problem by training a recurrent neural network (RNN) to perform the desired function. By developing a systematic perturbative method to interrogate the successfully trained RNNs, we are able to distill the underlying regulatory network among the biological elements (genes, proteins, etc.). Furthermore, we show several cases where the regulation networks found by RNN can achieve the desired biological function when its edges are expressed by more realistic response functions, such as the Hill-function. This method can be used to link topology and function by helping uncover the regulation logic and network topology for complex tasks.

## Abstract (web summary)

Networks are useful ways to describe interactions between molecules in a cell, but predicting the real topology of large networks can be challenging. Here, the authors use deep learning to predict the topology of networks that perform biologically-plausible functions.

## Introduction

Biological functions are carried out by the interaction of genes and proteins. The mapping between the interaction network topology and its function is one of the central themes in biology. Searching computationally for possible regulation networks that will give rise to a certain biological function has been an active and important area in systems biology. Such an approach may provide unified mechanistic understandings, help uncover and interpret natural regulation networks, as well as suggest new designs for artificial synthetic circuits.

In simple cases, where the network size being studied is small (~3 nodes) and the functional requirements can be abstracted to simple mathematical descriptions, functional network topologies can be found via exhaustive search^1–6^. Although the exhaustive search scheme has been fruitful in dealing with small network modules and functions with relatively low complexity, it faces fundamental challenges in scaling up to larger and more complex systems—the search space for network topology increases exponentially with the network size, which could easily reach the limit of the available computation power even for four-node networks. In some extension of the enumeration method, regulatory dynamics are modeled by Logistic regression (equivalent to single-layer artificial neural network, i.e., linear model plus sigmoidal saturation)^7,8^. This formulation brings remarkable improvement for parameter sampling, but in general, the computational difficulty of brutal-force enumeration still exists. Another extension is using knowledge from small modules to construct larger networks^6,9^, but such a constructionist approach requires certain prior knowledge of the building blocks and can only explore a very limited subset for larger networks. There exist other searching schemes under the trail-and-error spirit, for example, the in-silico evolution^10–12^, in which mutation and selection are repeatedly performed on the network structure to optimize a target fitness function. However, the applicability of the method is very much dependent on the fitness landscape and the implementation of the algorithm.

On the other side of the spectrum, if the biological function is described by large amount of gene expression data, the regulation network structure can be obtained through statistical regression methods, known as the task of network inference^13,14^. However, large amounts of data are usually needed to make those data-driven methods work. If only sparse and incomplete descriptions of the target function is available, finding the most probable trajectory (i.e., interpolating the data) is itself a hard task.

**Fig. 1:**
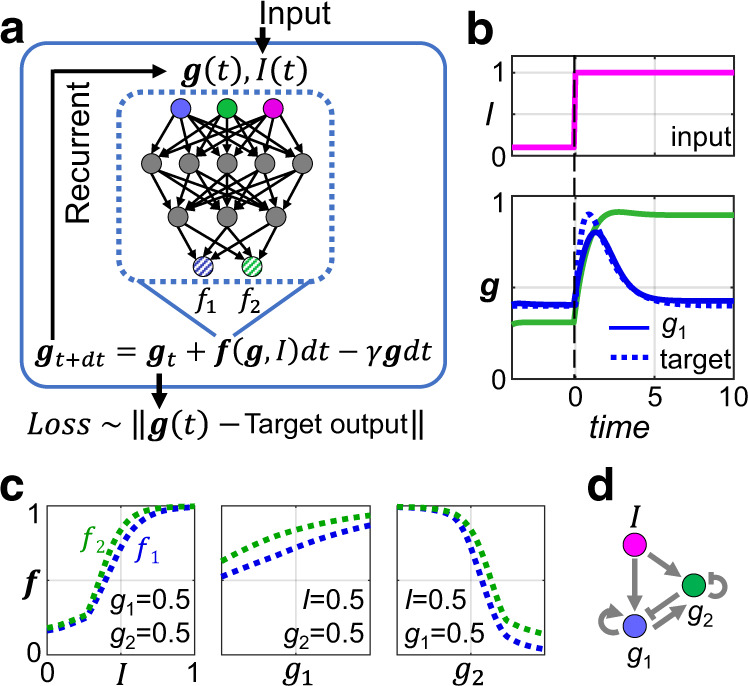
Method demonstration with adaptation of a two-node network. **a** The functional dependency of the synthesis terms *f*_1_ and *f*_2_ for *g*_1_ and *g*_2_ are evaluated by a small feed-forward NN (dashed box). *f*_1_ and *f*_2_ (shaded blue and green circles) can depend on all the three variables: *g*_1_, *g*_2_ and the input signal *I*. Time evolution of the dynamic system corresponds to recurrent iteration of the NN block. The output ***g***(*t*) = (*g*_1_(*t*), *g*_2_(*t*)) is compared with the target value to define the Loss function for training. **b** We require *g*_1_ to be adaptive to the input change (pink line) and set its target time-course values as indicated by the blue dotted line. No constraints on *g*_2_ are imposed. Blue and green solid lines are the time evolution of *g*_1_ and *g*_2_, respectively, after training. **c** *f*_1_ (blue dotted line) and *f*_2_ (green dotted line) as functions of *I*, *g*_1_ and *g*_2_, after training. The three panels show their dependence on *I*, *g*_1_ or *g*_2_ with the other two variables fixed. In all three subpanels, the horizontal and vertical axis both ranges from 0 to 1. **d** The regulation network drawn from the information of (**c**). For example, the first panel of (**c**) indicates that *f*_1_ increases with *I*, implying *I* activates *g*_1_.

We reason that the search for functional network structures (topologies) may be carried out more efficiently by employing deep artificial neural networks (NN). Firstly, unlike the enumeration or evolution approaches, which rely on trails and errors to find a satisfactory network topology, training the deep neural network (DNN)^15,16^ is a more targeted process. NN rewires itself directly using the information propagated back from fitting. Secondly, NN-based models can still be successfully trained even the target dynamics are only partially observable (i.e., with limited data on some genes at some time points). To be specific, in our approach the gene regulation network (time-evolution-function) is represented by a feed-forward NN. Numerical integration of the dynamic system corresponds to stacking this feed-forward module into a recurrent neural network (RNN), which can be trained efficiently by backpropagation if the desired biological function has been properly formulated as a loss function (Fig. 1a).

The idea of learning differential equations from data has attracted much attention recently, and there have been many successful attempts in this direction^17–20^. These studies are mainly focused on developing the mathematical method itself, especially on finding efficient ways for training an accurate NN simulator of the underlying unidentified dynamical system. In this paper our aim is to find the underlying regulation network(s) for a given biological function. Thus, we not only have to train the RNN, but more importantly also to interpret it biochemically, by performing sensitivity analysis^21,22^, to establish a connection between the trained RNN simulator and the more traditional description of gene regulation network.

Besides training and interpreting the RNN, we also put effort in validating whether the networks obtained are biologically feasible. DNNs have the potential of overfitting. For our application here, there are possibilities that the RNN relies on some highly non-monotonic forms of regulations that no biochemical system can achieve. We tackle this problem by verifying whether the regulation network found by RNN can still achieve the desired biological function after its links being expressed by Hill-functions (HF).

In this paper, we demonstrate our methods with four bio-inspired examples, adaptation, controlled oscillation, pattern formation and a set of 10-node cellular automata. RNNs can be trained to achieve all functions easily. After training, an in silico mutation method is applied to obtain a biochemically meaningful regulation network, which describes what the RNN learns. (This technique is also extended to sparsen the effective regulation network, thus help to seek for minimal functional modules). For the resulting network topologies, we can explain intuitively how their structures could give rise to their functions, compare them against existing biological networks, as well as demonstrate that many of them can still achieve the target function if being cast into more traditional HF models.

## Results

### Idea demonstration with adaptation

We first demonstrate the basic idea and the implementation of our method with adaptation, a simple, ubiquitous, and well-studied cellular function. In this task, the output node should sense the change of the input stimulus and then return to its pre-stimuli level even the stimulus persists (Fig. 1b). Regulatory networks (with no more than three nodes) for this function have been exhaustively studied^3,23^. Only two genes $\left({g}_{1},{g}_{2}\right)\equiv {\boldsymbol{g}}$ plus one input signal *I* are considered here for simplicity. Here, $f\equiv \left({f}_{1},{f}_{2}\right)$ represents the synthesis rate of the genes.

1 $$\frac{d{g}_{i}}{{dt}}={f}_{i}\left({g}_{1},{g}_{2},I\right)-\gamma {g}_{i};\,i=1,2$$

The function ${\boldsymbol{f}}\left({\boldsymbol{g}},{I}\right)$ contains information about gene-gene interactions, which is usually written in explicit formulas but is now simulated by a small feed-forward NN (Fig. 1a). This feed-forward NN uses the current value of ${\boldsymbol{g}}\left(t\right)$ and $I\left(t\right)$ as its input and generates ***f*** as its output, thereby computes ${\boldsymbol{g}}\left(t+{dt}\right)$. So, this is a kind of NN-based auto-regressor. By defining ***f*** to be non-negative (between 0 and 1), and with the explicit liner degradation terms $-\gamma {g}_{i}$, our formulation automatically prevents ***g*** from diverging. (We simply set $\gamma =1$, since degradation in reality can also be accounted by diagonal terms in ***f***). Time evolution of the regulatory system then corresponds to iteration of this NN block, yielding an RNN model. Note that while a full-fledged RNN passes hidden-layer information from each time point to the next, in our case, only observables ${\boldsymbol{g}}$ are passed from *t* to *t + dt*.

The Euclidian distance between model trajectory and the target response curve $\hat{g}$ is used as Loss function for training. Here, we require the output node (${g}_{1}$) to carry out the adaptation function (Fig. 1b). The other node (${g}_{2}$) has no functional constraints and can play a regulatory role. Therefore, ${Loss}=\sqrt{\mathop{\sum}\limits_{t}{\left({g}_{1}\left(t\right)-{\hat{g}}_{1}\left(t\right)\right)}^{2}}$. With a step-like input signal *I*(*t*), the target response curve ${\hat{g}}_{1}$ should in general be pulse-like—having a fast response phase and a slower recover phase. Any curve with this kind of shape can serve as the training target, and that used in Fig. 1 is simply defined as the sum of two exponentials. (See Fig. S1a–c for another case). Training converges quickly, yielding perfect adaptation (Fig. 1b)—that is, a negligible adaptation error (difference between the pre-stimulus and the fully adapted *g*_1_ levels) as well as high sensitivity (response peak).

For this low-dimensional system, the trained ***f*** function can be plotted directly (see Fig. 1c for typical cross-sections and Supplementary Fig. 1d for the entire surface). Note that the ***f*** function is rather smooth and monotonic, indicating that the RNN is not overfitting at least in the narrowest sense. By observing whether ${f}_{1}$ and ${f}_{2}$ are increasing or decreasing with ${g}_{1}$, ${g}_{2}$, and input *I* (i.e., sign of the partial derivative), one can easily find the regulatory logic hidden in the trained RNN. For example, the fact that ${f}_{1}$ increases with *I* (Fig. 1c, left panel) implies that *I* activates ${g}_{1}$. The underlying regulation network adopted by the RNN can thus be constructed (Fig. 1d).

The network consists of both an incoherent feed-forward loop and a feedback loop, both known as the elementary motifs for adaptation^3,23^. Intuitively, this small network works as ${g}_{1}$ is first activated by *I*, and later repressed by ${g}_{2}$ after ${g}_{2}$ reaches to a functional level.

### The in-silico mutation method: uncover learnt regulations and guide training

For systems with more genes, direct visualization the ***f*** function may be difficult. In this case, one could use the partial derivative $\partial {f}_{j}/\partial {g}_{i}$ to reflect the regulation effect of ${g}_{i}$ on ${g}_{j}$. Theoretically, this provides an effective way to reveal the learnt regulation network without having to read the high-dimensional NN weights. But two points need to be discussed to make it actually work. First, it matters where these derivatives are evaluated, because only parts of the phase-space region $\left({\boldsymbol{g}},{I}\right)$ are relevant to the task on which the NN are properly trained. Therefore, one should evaluate the derivatives near the wild-type trajectories (corresponding to $\lambda \approx 1$ as discussed below. See also Supplemental text S1). Second, magnitude of the derivative by itself may not be an accurate representation of the regulation strength. Imagine the case that ${g}_{j}$ synthesis is strongly repressed by a high-expressing gene ${g}_{i}$ . Since ${f}_{j}\approx 0$, $\left|\partial {f}_{j}/\partial {g}_{i}\right|$ does not tend to be large, although in this case ${g}_{i}$ is actually the main repressor of ${g}_{j}$. Thus, a more accurate measure would be the change of ${f}_{j}$ upon the fold change of ${g}_{i}$.

2 $${\Delta }_{{ij}}\equiv {f}_{j}\left(\cdots ,{g}_{i}\right)-{f}_{j}\left(\cdots ,{\lambda g}_{i}\right);\,0 \,<\, \lambda \,<\, 1$$

This definition is reminiscent of the knockdown experiments biologically. The discount factor *λ* controls the magnitude of the perturbation—zero means link-knockout, i.e., deleting the binding sites of transcription factor *i* on the regulatory region of gene *j*, while a close-to-one value yields kind of “knockdown derivative” of the regulation link *ij*. In Table S2 we compare the results with different values of *λ*. Smaller perturbations ($\lambda \,> \, 0.9$) work better in general. If averaged along a WT trajectory, ${\langle {\Delta }_{{ij}}\rangle }_{{WT}}$ quantifies the effective gene regulation from ${g}_{i}$ to ${g}_{j}$. (This is the implementation used later in Figs. 5 and 6).

An alternative and maybe biologically more direct way to reveal the regulation is to simulate the dynamics of the link-knockdown/knockout mutant with the learnt ***f*** function. For example, the mutant trajectory with the regulatory link from ${g}_{1}$ to ${g}_{2}$ being knocked down is given by:

3 $$\left\{\begin{array}{c}\frac{d{g}_{1}}{{dt}}={f}_{1}\left({g}_{1},{g}_{2},I\right)-\gamma {g}_{1}\\ \frac{d{g}_{2}}{{dt}}={f}_{2}\left(\lambda {g}_{1},{g}_{2},I\right)-\gamma {g}_{2}\end{array}\right.;\,0 \,<\, \lambda \,<\, 1.$$

**Fig. 2:**
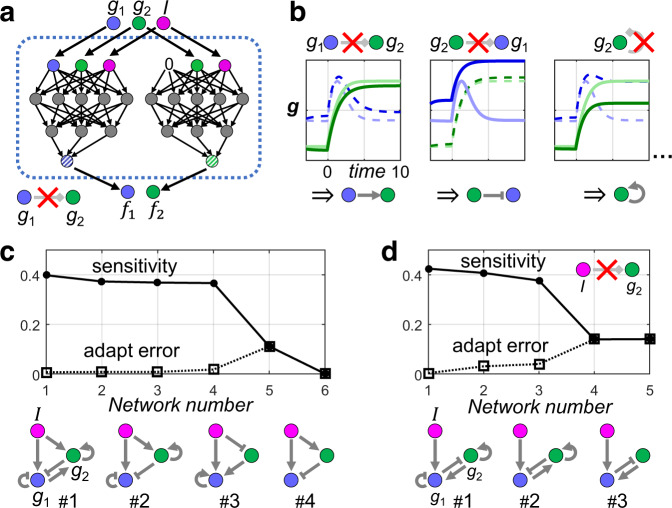
Manipulating RNN to simulate link-knockout mutation. **a** Method for blocking the regulation link from *g*_1_ to *g*_2_, i.e., *g*_1_ is set to 0 when computing *f*_2_. **b** This perturbed ***f*** function can be iterated to simulate the effect of the mutant in which a specific regulation link is deleted. For example, with an RNN trained on the adaptation task, blocking the regulatory effect of *g*_1_ on *g*_2_ results in an increase in *g*_2_ (from lighter to darker solid green line), indicating a self-inhibition (left panel). Difference in the *g*_1_ level is not important here (dashed lines). Similar arguments apply to the other two panels. Summarizing this information gives a regulation network shown as topology #1 in (**c**), bottom left. **c** Sparsening the regulation network iteratively by removing non-necessary links sequentially, as described in the main text. The upper panel plots the sensitivity and adaptation error for the sequence of networks. The lower panels show the network topologies at steps 1 to 4. The minimum incoherent feed forward motif appears naturally (topology #4), before the network has too few links to adapt. **d** With the regulation from *I* to *g*_2_ blocked at the very begging, similar procedures as in (**c**) would find the minimum negative feedback loop, the other core motif for adaptation besides the incoherent feed forward loop.

The regulation logic of the underlying gene network can then be obtained by comparing the resulting dynamics with that of the WT trajectory. An increase in ${g}_{2}$ level in this mutant would imply a negative regulation or inhibition of ${g}_{2}$ by ${g}_{1}$, and vice versa. Figure 2b shows three examples of such link-knockout study (i.e., $\lambda =1$). For example, in first panel, upon blocking the interaction from ${g}_{1}$ to ${f}_{2}$, the level of ${g}_{2}$ drops slightly (from the lighter to the darker green line), indicating activation of ${g}_{2}$ by ${g}_{1}$. Performing such link mutation on each and all possible interactions yield the regulation network shown as topology #1 in the lower panel of Fig. 2c, which is identical to that obtained by directly plotting the ***f*** function in this case (Fig. 1d).

In the $\lambda \to 1$ limit, the mutant trajectory approaches that of the WT, thus the link-knockdown study becomes equivalent to computing the partial derivative (Eq. 2). However, the knockdown (or knockout) trajectory introduced here can also be used in another way—to search for sparse or minimal networks as discussed below.

The network shown as topology #1 in Fig. 2c contains both of the elementary adaptation motifs: negative feedback loop and incoherent feed-forward loop, any one of which suffices to achieve adaptation^3^. This kind of redundancy is typical for the regulation network learned by RNN without any structural constraints. RNN simply searches for a time-iteration rule that works rather than being minimal or sparse. Simple regularizations like weight decay do make the ***f*** function smoother, but help little in sparsening the effective regulation network, as NN parameters do not have explicit correspondence to effective regulatory links (Supplementary Fig. 2, and Supplemental text S2 for an different regularization attempt).

Efficient reduction of the redundant or undesirable links can be achieved by implementing the link mutation technique (Fig. 2a) before training. The RNN is thus constrained to find solutions without certain regulatory links. Since in most situations the truly necessary links are not known a priori, an iteration of the RNN-based model is needed to find sparse core networks. Starting with a redundantly connected solution (#1 in Fig. 2c), apply the link-mutation test for every existing link, find the one that has minimal phenotype change upon deletion, then retrain the model with this link deleted, and iterate. This idea is somewhat similar to learning both NN architecture and weights through training^24^. In this way, a sequence of regulation networks with a decreasing number of links can be obtained. The minimum incoherent feed-forward network emerges (#4 in Fig. 2c) before the network has too few links to achieve adaptation.

The same iteration procedure can also be carried out with the constraint that input *I* should act only on the output node ${g}_{1}$: deleting the link from *I* to ${g}_{2}$ at the very beginning. The incoherent feed-forward structure is now impossible, and the minimum negative feedback loop emerges (Fig. 2d).

### Controlled oscillation

**Fig. 3:**
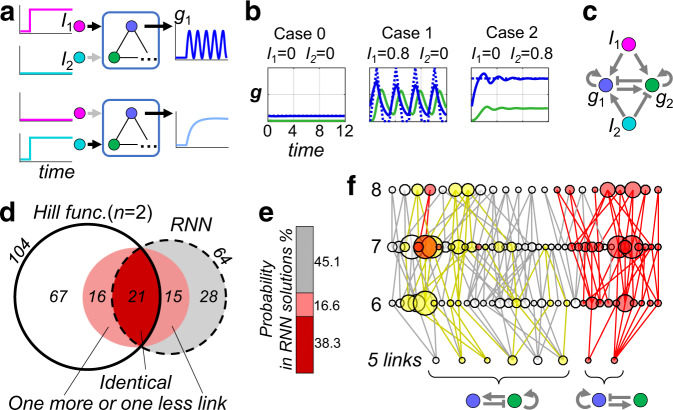
Systematic study of RNN-discovered network topologies with the controlled oscillation task. **a** Task description. The regulatory system is expected to generate an output (node *g*_1_, blue) that shows oscillatory response to input stimulus *I*_1_ (magenta) and steady-state response to *I*_2_ (cyan). **b** Training the RNN model. The training target consists of three parts, corresponding to resting state without stimuli, oscillation under *I*_1_ (*I*_1_ = 0.8, *I*_2_ = 0), and sustained high expression under *I*_2_ (*I*_2_ = 0.8, *I*_1_ = 0). Target values for *g*_1_ are shown as dotted blue lines, and can be well fitted by the RNN after training (solid lines). No constraints on *g*_2_ are imposed. All links are allowed during training without any special constrains. **c** The underlying regulation network of the trained RNN, obtained by the link-mutation method introduced previously. It can also be successfully transferred to a Hill-function model (Fig. S3b). **d** Venn diagram for network topologies found by RNN and Hill-function-based enumeration. A total of 64 topologies were found by 200 repeated RNN trainings (dashed circle); and 104 topologies were obtained by Hill function (HF) based exhaustive search (solid circle). 21 of them are identical (dark red region). Another 15 RNN discovered topologies differ from the HF topologies only by one more or one less link, thus having the same core topology. They are drawn “near” the strictly overlapping region (lighter red region). Similar situation also occurs on the HF side. **e** Regarding the probability of occurrence, the 21 direct hit topologies account for 38% of total probability; and 55% for all those HF-compatible ones. **f** The search bias of RNN. And, this bias can be changed via different training settings. Each successful network found by HF-based exhaustive search is represented by a dot whose size reflects the chance of finding a successful parameter set with random sampling. They are arranged in four layers according to the number of regulatory links they have (5, 6, 7, and 8 from bottom to up, respectively). Structurally similar ones are connected by lines. Most RNN-relevant topologies (red part in (**d**)) lies on the right-hand-side branch. However, by modifying the detailed training settings (see main text and Supplemental text S6), RNN can be pushed to explore the left-hand-side branch (yellow).

Our second example is controlled oscillation. That is, the same core regulatory module exhibits qualitatively distinct dynamic behaviors under different kinds of external stimuli^25^—oscillatory response to input ${I}_{1}$ and steady-state response to ${I}_{1}$ (Fig. 3a). With this example, we will first demonstrate the applicability of the above introduced methods, and systematically compare the network topologies found by RNN with those found through the exhaustive search.

The task is defined as follows. The output node ${g}_{1}$ should have: 1) low basal level in the absence of both stimuli, 2) oscillation when input ${I}_{1}$ is present but not ${I}_{2}$, and 3) sustained high expression when ${I}_{2}$ exists but not ${I}_{1}$ (dashed lines in Fig. 3b). Precise values of oscillation frequency, response plateau, etc., as well as the oscillation waveform (set as triangular here) does not affect the resulting network topology in general. In Fig. 3b, the solid lines show a trained example of a two-gene network. Fig. 3c shows its underlying regulation logic, revealed using the link-mutation method. It contains a negative feedback loop responsible for the oscillation. Input ${I}_{1}$ activates the feedback module hence induces oscillation. Input ${I}_{2}$ also activates ${g}_{1}$ but at the same time represses ${g}_{2}$, preventing it from being activated later by ${g}_{1}$, therefore the negative feedback gets shut-off in the high- ${g}_{1}$ state.

### Implementing the RNN-discovered regulation network with Hill-function model

As has demonstrated, training an RNN to perform some biological tasks, and mapping it to a regulation network can be carried out in a straightforward manner. However, it is important to discuss the issue of overfitting as DNNs may simply fit everything^26^. In the context of biological regulation networks, we need to verify whether the resulting networks are biologically implementable, not relying on some highly non-monotonic input-output functions that are unreasonable biochemically.

It is hard to strictly define whether a proposed network topology is biologically implementable. Here, as a rough evaluation, we check the consistency of the RNN-based model with a biochemically interpretable model where the term ***f*** in Eq. 1 is represented by the Hill function (HF), which is widely used for modelling biological regulations. (See Methods for our detailed implementation). Specifically, we will verify whether the regulation network found by RNN can still achieve the desired function if being converted to an HF-based model. Note that this test is partial at best—while HFs do represent a class of biologically realizable regulations, they certainly do not cover all kinds of bioregulation. As expected, HF-consistency also correlates with input-output monotonicity (Fig. S3e). As shown in Fig. S3b, the network in Fig. 3c can be successfully converted to HF models, and so do the two basic adaptation networks in Fig. 2c, d^23^.

### Systematic comparison between RNN Model and Hill function-based model

To make a systematic comparison, we first performed an exhaustive search using the HF model. Among a total of 2304 possible network topologies, 104 can achieve the controlled oscillation task under HF model. Also, we train the RNN model repeatedly 200 times, with different weight initialization and run-time Langevin noise. When identifying network topologies found by RNN with the link-mutation method, some regulation links are extremely weak, so a cutoff is certainly needed to decide whether a weak link can be regarded as non-existent. Without a cutoff, topologies with fewer links may become indistinguishable with other denser ones; on the other hand, if the cutoff value is too high, topologies with more links would be missed. So, there exists an optimal cutoff value that gives a maximum number of distinct network topologies (Fig. S3c), which is used here.

A first observation is that RNN solutions are highly degenerated. Although the 200 trained RNNs have quite different final weight values, only 64 different effective regulation networks emerged. Furthermore, the most frequently occurring 30% topologies take up 80% of the total probability.

Among the 64 topologies obtained, 21 appear in our HF-based exhaustive search result, and another 15 differ from the HF topologies only by one more or one less link (Fig. 3d). As adding or removing a link will not change the core network topology, these 15 solutions should also be regarded as “HF-compatible”. Regarding the probability of occurrence, the 21 direct-hit topologies account for 38.3% of total probability; and 54.9% for all those HF-compatible ones (Fig. 3e), which is rather high. As a comparison, a total of 2304 topologies that satisfy the basic connectivity requirements are studied in the enumeration, of which only 104 (about 4.5%) are successful.

On the other hand, those RNN-discovered topologies (21 + 16 = 37 in total) are not evenly distributed among the total 104 topologies obtained by HF-based exhaustive search. Following the visualization scheme adapted from ref. ^7^, we organize all these 104 HF topologies into a graph, according to the number of regulatory links and their structural similarity (Fig. 3f). Each topology is represented by a node, whose size reflects its robustness with HF model (the number of valid parameter sets found in 160,000 random samples). Their structural similarities are shown by connecting pairs of them by lines if they differ from each other by only one regulatory link. By coloring those RNN-discovered topologies in red, it is clear that they span mainly the right-hand-side branch. A closer investigation reveals their common core module – ${g}_{1}$ activates both itself and ${g}_{2}$, while ${g}_{2}$ represses ${g}_{1}$. This is the most robust two-node oscillatory module according to enumerative study^5^. But the left branch is largely unexplored under the current training setting. RNN seems to have its own bias toward different feasible regulation structures.

Such bias of RNN model is not unchangeable—it can be pushed to explore the left branch if its detailed implementation were modified. For example, we can set the pre-stimulus level of ${g}_{2}$ to a fixed high value 0.9 (while in original settings it is a free parameter), and the RNN is first trained to perform unconditional oscillation and later the full controlled oscillation task (Supplemental text S5). With these modifications, the RNN becomes biased towards the left branch (yellow, in Fig. 3f).

### Searching for sparse controlled-oscillation-networks by sequential removal of regulation links

**Fig. 4:**
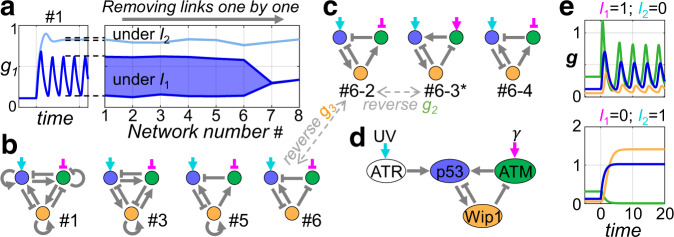
Searching sparse three-node network topologies for controlled oscillation task. **a** The method in Fig. 2c, d is applied to the controlled oscillation task. The resulting networks with decreasing number of links are labeled by numbers 1 to 8. The darker and lighter blue regions show the range of oscillation and level of steady state response under *I*_1_ and *I*_2_, respectively, after training. Networks #7 and #8 are too sparse to be successfully trained. **b** Effective regulation networks mapped out from this sequence of models. **c** Possible branching at step #6 for panel (**b**). Several repeats of this step give another three types of topology #6: named #6-2, #6-3, and #6-4, respectively. Topology #6, #6-2, and #6-3 share a common underlying structure. Just reversing the sign of certain nodes would make them identical (dashed gray arrows). **d** Topology #6-3 (marked by asterisk in panel **c**) is the same as the core oscillation module of p53 network. This is a well-known example for controlled oscillation. This panel is adapted from ref. ^27^ but another node Mdm2 is not included. **e** Topology #6-3 in panel (**c**) can be successfully transferred to Hill-Function models.

The method for searching possible minimal networks presented in Fig. 2c, d is also applied to the controlled oscillation task. Here, we will search for sparse three-node networks as they have more redundant links to be removed than two-node ones. Detailed implementation is not much differed from the previous adaptation example. A sequence of regulation networks with decreasing number of links are obtained in this manner (Fig. 4a, b). The task can be successfully trained for network #1 throughout #6.

Repeating the above training-and-deletion sequence several times result in different sparse regulation network solutions. See Fig. S5 for another example. Indeed, several repeated training with the allowed links of #6 yield another three possibilities of its topology (Fig. 4c). Among them, topologies #6-2 and #6-3 share a common underlying high-level structure with #6. Reversing the sign of ${g}_{3}$ in #6 (swapping activation and inhibition for all regulations connecting to ${g}_{3}$, as well as reversing its own expression level) will turn it into topology #6-2. Similarly, #6-3 is equivalent to #6-2 if ${g}_{2}$ were reversed in this way. Interestingly, the topology #6-3 is identical to the core p53 oscillation module, a well-known example for controlled oscillation (Fig. 4d, adapted from ref. ^27^, but Mdm2 is not included). Also, as expected, this topology (#6-3) can be successfully transferred to HF models (Fig. 4e and Table S4).

### Gap gene patterning

In the next example, we demonstrate our method on a more complex case using real data from experiments—the spatial patterning of gap-gene expression in *Drosophila* embryogenesis. This is a well-studied system both experimentally and by modelling^28,29^. Here, the RNN model is used to solve the inverse (reverse-engineering) problem: given the observed gap gene expression pattern as the desired output, find possible underlying regulation networks. The results are further compared with the known biological network obtained from decades of experimental studies.

**Fig. 5:**
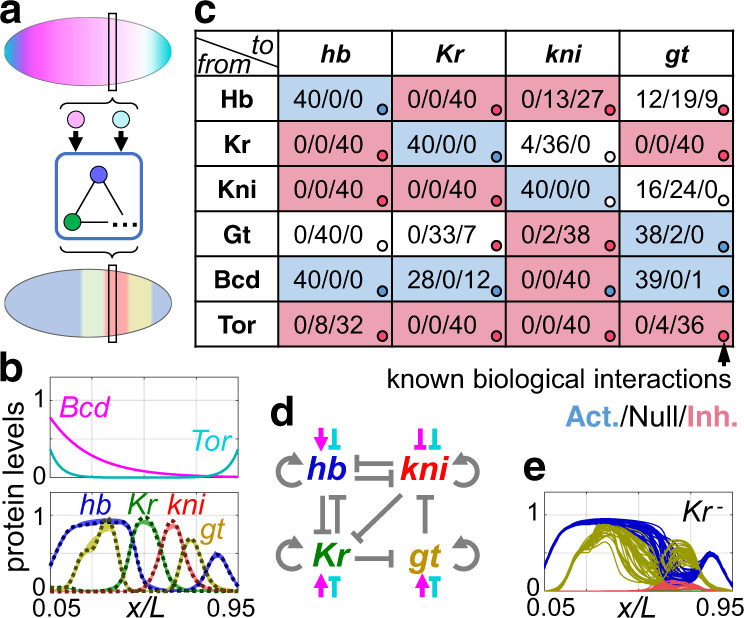
Train RNN to generate the gap gene pattern. **a** At each spatial point, the gap genes interact with each other under the inputs of maternal morphogens, generating stripe-like patterns along the *Drosophila* Anterior-Posterior body axis (anterior to the left and posterior to the right, same for **b**). The RNN is trained to simulate the gap gene network to generate the given output pattern. **b** Upper: the morphogen profiles, serving as static inputs (Bcd: Bicoid, Tor: Torso). Lower: the target gap gene profiles (dotted lines, cited from the FlyEX database^30^) and the patterns generated by RNN after training (colored solid lines). Blue, green, red and yellow lines stand for *hunchback*(*hb*), *Krüppel*(*Kr*), *knirps*(*kni*) and *giant*(*gt*) expressions, respectively. Results of 40 parallel trainings are shown. **c** Statistics of the underlying regulation network of these 40 RNN solutions. For each of the regulatory link, the frequencies of this link being activating/non-exist/inhibiting are shown, and the block is colored following the majority (blue/white/red for activation/non-exist/inhibition). Colored dots on the lower right corner represent the biological gap gene interactions revealed by experiments, which is very similar to the majority network learnt by RNN. **d** The majority network found by RNN, same as the colored blocks in (**c**). **e** The similarity between the RNN-discovered network and the biological one is further illustrated in the *Kr* mutant. Being negatively regulated by Kr, both *hb* and *gt* domains expand thus eliminate the *kni* domain. This prediction is consistent with experimental observations[31].

Roughly speaking, four gap genes (*hb*, *Kr*, *kni*, *gt*) respond to the input signals provided by maternal morphogen gradients (Bcd and Tor), forming band-like expression patterns along the Anterior-Posterior (A-P) axis (Fig. 5a). Though spatial degree of freedom (A-P axis) is involved, our model here is not spatially coupled – the effect of short-range diffusion of gap gene products is neglected. Thus at every single spatial position the gene regulation is modeled by an RNN following Fig. 1a, with ${\boldsymbol{g}}$ and ${\boldsymbol{f}}$ both being four dimensional vectors (*hb*, *Kr*, *kni*, *gt*), and the input ${\boldsymbol{I}}$ has two components (Bcd, Tor). Target expression pattern is a typical pattern (at a single timepoint) taken from the FlyEX database^30^, and the morphogen gradients are treated to be static. No time series data is used for training. The RNN runs freely for several steps and is compared with this target profile at the final time point, giving the Loss function for training.

The RNN can be easily trained to generate this pattern accurately. Results of 40 repetitive trainings all overlap with the target pattern (Fig. 5b, lower). When being mapped out to effective regulation networks using the link-mutation method, all of the 40 trained RNNs turned out to have similar effective structures. In Fig. 5c, each regulatory link is represented by a block, in which the frequencies of this link being activating/non-exist/inhibiting among the 40 solutions are listed, and is colored following the majority (blue for activation and red for inhibition). This majority network (redrawn as Fig. 5d) has very similar structure as the known biological gap gene network revealed by experiments^28^. The latter is represented in Fig. 5c by colored dots in the lower-right corner. Such similarity between the RNN-discovered gap gene network and the biological one is further illustrated by the *Kr* mutant. As both *hb* and *gt* are inhibited by Kr, in *Kr* mutant these domains expand towards the center, eliminating the *kni* domain through repression (Fig. 5e). These features are consistent with experimental observations^31^. We would not claim that our approach provides a better model for the *Drosophila* gap gene system than the one obtained by decades of experimental work on many mutants. Instead, this example helps to demonstrate the capability and flexibility of our approach for reverse-engineering more complex networks from very limited experimental data.

### Continuous-state cellular automata in 10-dimensional state space

The final example deals with a much larger regulation network (10 nodes) with much more complex spatial-temporal behaviors. The RNN models are trained to simulate the dynamics generated by some in silico ground truth regulation networks, and are used to predict the underlying activating/ inhibiting gene interactions (through the link-mutation method).

**Fig. 6:**
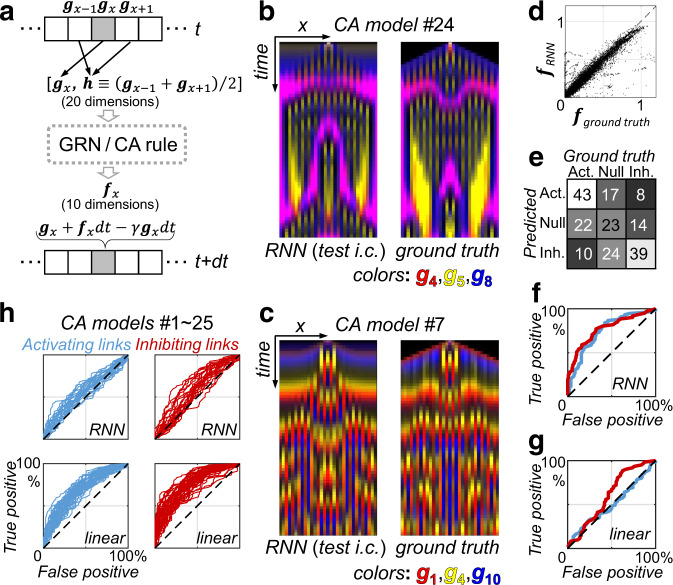
Uncovering regulatory links in 10-node continuous-state cellular automata. **a** Illustration of the cellular automata (CA) system. Expression levels of 10 genes define the state of each cell. The cells are arranged in a 1-dimensional array, and at each time step, each cell updates its state according to its current state (***g***) as well as the average of its nearest neighbors (***h***). We first implement the regulation network, or CA rule, by Hill functions to generate “ground truth” CA models, then use the time-course data generated by them to train the RNN. Periodic boundary condition is used. **b** After training, the RNN model simulates the underlying dynamical system accurately. Starting from an unforeseen initial condition, the RNN model (left) generate spatial-temporal dynamics quite similar to that generated by the ground truth model (right). Only three genes that varies significantly are shown as red, yellow and blue here. **c** Another case just like (**b**). **d** A set of ***g*** values (as well as that of neighboring cells) are generated by the ground truth CA model #24 (that of panel **b**) starting from a set of random initial conditions. With these same set of input, the ***f*** term computed by RNN ***f***_RNN#24_ (***g***, ***h***) is plot against that given by the ground truth HF model ***f***_ground truth_ (***g***, ***h***). **e** For the RNN model of panel (**b**), the weakest 30% of links revealed by the link mutation study is predicted to be non-exist, and the remaining negative/positive ones are classified as inhibiting/activating. The confusion matrix of such a link-type classifier is shown. More than half of existing links are correctly predicted. **f** The link mutation results can also be used to define a binary classifier, which distinguishes activating links against non-activating ones (non-exist or inhibiting). The receiver operating characteristic (ROC) curve of this activating link predictor is shown in blue (for CA #24). Similar situation applies to distinguishing inhibiting links as well (red). Our RNN based models significantly out performs the simplest linear auto-regressor, whose ROC curves under the same definition are shown in (**g**). This feature holds for all 25 different CA models studied (**h**).

The spatially coupled dynamical systems, serving as ground truth here, is a kind of continuous-state cellular automata (CA) (Fig. 6a and Supplemental text S10). CA is widely used in modelling complex interacting systems^32^. Here, the gene regulation network shared by all cells computes the synthesis rate (***f***, 10 component) of each gene within the current cell using the current expression state of itself (***g***) as well as that of its neighboring cells (***h***). Here, we study only regulatory rules with reflection symmetry, hence defining ${{\boldsymbol{h}}}_{x}=\left({{\boldsymbol{g}}}_{x-1}+{{\boldsymbol{g}}}_{x+1}\right)/2$ where *x* labels cell position. Therefore, the regulation network (or CA-rule) have 200 possible regulatory links. The ground truth networks are first generated at random; each link has 35% probability to be activating or inhibiting respectively and 30% probability to be non-existent, and then is converted to HF models with randomly-sampled parameters. Those CA-rules that display non-uniform spatial-temporal patterns are selected as ground truth networks.

Spatial-temporal data generated by the ground truth network is used to train the RNN model. As demonstrated by the previous examples that our method does not rely on detailed time-series data, we down sampled the spatial-temporal profiles for 10-fold along the temporal direction to prepare the training data, (i.e., the CA dynamics is observed every 10 timesteps). The RNN model starts with an observed state, run freely for 10 timesteps to predict the next one. Even for this much more complex case, RNN training converges fast (~7 min on a 4-core laptop).

Since CA dynamics are in general chaotic, it is natural that different initial conditions should give rise to different spatial temporal patterns. To see if the trained RNN has some predictive power, it is tested on some random new initial conditions—it runs freely after initialization to generate the entire predicted pattern, which is compared to that generated by the ground truth network with the same new initial condition (Fig. 6b, c). Although the two dynamic trajectories diverge after long enough time, the RNN model has nonetheless captured the main spatial-temporal features. For the ***g*** values along a test CA trajectory, the ***f*** term computed by RNN is in most cases close to that given by the ground truth HF model (Fig. 6d). The RNN is indeed a good approximation to the underlying regulation function.

For each regulatory link, the RNN model (with the link-mutation method) give a scalar-valued evaluation of its sign and strength. In predicting the underlying network topology, we discard the weakest 30% links, and the remaining positive and negative ones are predicted as activation and inhibiting, respectively. Figure 6e shows the counts of correct and incorrect predictions for CA-model #24 following this definition (the case in Fig. 6b). The accuracy is fairly good. For example, there are a total of 61 true inhibiting links in this case, and 39 of them are correctly predicted.

On the other hand, the strength threshold for identifying an activating link against non-existing or inhibiting ones can be tuned continuously to give a receiver operating characteristic (ROC) curve (Fig. 6f for CA-model #24). The same applies to distinguishing inhibiting links. Note that our RNN model, which is effectively an auto-regressive model, significantly out performs the simple and widely used linear auto-regressor (whose ROC on CA-model #24 is presented in Fig. 6g). The RNN-based method has similar performances on all 25 different ground truth CA models studied (Figs. 6h and S6).

## Discussion

In this paper, we explored the possibility of searching feasible regulation networks behind given biological functions (represented by the expression level of one or more genes) through training and interpreting RNN. RNN is first trained to perform the biological function (with or without some external constraints). Then, a link-mutation method is introduced to interrogate it to give an effective underlying regulation network. Different aspects of our general approach are demonstrated with four examples.

There are multiple advantages of simulating unidentified dynamical systems with RNN. Firstly, model fitting is accelerated greatly by using RNN and backpropagation. (Table S1 lists the time requirements of RNN training versus random parameter sampling for the cases studied in this paper.) Secondly, the RNN model is demonstrated to be effective even if there are only sparse descriptions of the target biological functions. In our first two examples (Figs. 1–4), only the target response curves on a single gene is provided for training. And in the next two examples (Figs. 5 and 6), RNN can still be successfully trained without or with only down-sampled time series data. Thirdly, multilayer NNs have a less rigid form for the input-output function than predefined equations. Training can be viewed as establishing the necessary logical information connections between the input and output nodes, without being restricted by details in conventional modeling approach such as the way to describe cooperativity, the form of nonlinearity, etc. Our implementation of time integration in this paper is carried out by simple forward Euler method (Fig. 1a). For more demanding tasks, more advanced numerical framework like the neural-ODE^19^ can be used instead.

An important issue is whether the network topologies proposed by RNN can have biological realizations. Although about half of the RNN-discovered networks can pass a stringent consistency check using HF-based models, some unrealistic solutions do appear. However, these unrealistic (or kind of undesired alternative) solutions may be consequences of insufficient constraints in training, rather than unavoidable model limitations. Consider an example in the adaptation case. If only a single input strength (platform height of the step function) were used for training, in some RNN solutions adaptation is not achieved through appropriately structured regulation network but by some non-monotonic regulation function form – ${g}_{2}$ increases slowly due to activation from input *I*, and ${g}_{1}$ is activated by low concentration of ${g}_{2}$ and inhibited by high concentration of ${g}_{2}$, hence generating a pulse-like response. One can reformulate the loss function, or introduce additional training constraints, to avoid such undesirable solutions. For example, in our treatment of adaptation in Figs. 1 and 2 these solutions are eliminated by varying the input strength in training.

Another issue is that the networks proposed by RNN may bias towards certain sub-class in the solution space as discussed in Fig. 3f. As gradient descent (backpropagation) is a history-dependent searching algorithm, this bias should be largely determined by the training dynamics (hence, details of the model and Loss function). Although in principle RNN with a multi-layered recurrent module should be able to simulate all possible network topologies, the fact that it is trained through backpropagation actually makes it explore only a subset of the entire solution space given a specific implementation. Especially, as NN weights are usually initialized near zero to avoid gradient exploding, repeated trainings of NN tend to fall into the same degenerated minimum. One may explore other possibilities by using different implementations of the loss function, different detailed forms of target response curves, or introducing different regularization for RNN.

Finally, we note that the network topology may not be the sole determinant for function. For example, according to ODE-based network model study^5,33^, self-sustained oscillation should rely on delayed negative feedback loop. But this “rule” can be broken by including stochasticity^34^. Moreover, there exist more complex cellular processes that cannot be simply attributed to activating or inhibiting regulations. The concept and structure of regulatory network itself could be revisited in a broader context^35^. The exercise and lessons presented here may serve as a starting point for further exploration.

## Methods

### Training the RNN model

The NN simulators for simulating the synthesis rates ***f*** in this paper are basically multilayer perceptrons (MLP). For the results of Figs. 1, 5, and 6, a single MLP is used, with the input layer having the dimensionality of the number of genes plus the number of inputs (*N*_*gene*_ + *N*_*input*_); and output dimension *N*_*gene*_. Two hidden layers with equal numbers of nodes are used. The case of Figs. 2, 3, 4 are slightly different, where each output node has its private MLP (*N*_*gene*_ + *N*_*input*_ dimensional inputs and one-dimensional output), thus implementing the structure shown in Fig. 2a explicitly. A total of *N*_*gene*_ MLPs of this kind are used. Throughout this paper, *ReLU* is the activation function for the hidden layers, and for the output layer we use *sigmoid* to keep the output value (synthesis rate *f*) bounded between 0 and 1.

This MLP computes the synthesis term ***f***. The ***f*** term is then integrated through time to compute a time-course trajectory. Numerical integration follows the Euler method (*t = i dt*). Decay rate *γ* is set to 1.

4 $${\boldsymbol{g}}\left(i+1\right)=\left(1-\gamma {dt}\right){\boldsymbol{g}}\left(i\right)+{\boldsymbol{f}}\left({\boldsymbol{g}},I\right){dt}$$

By using a relatively large timestep *dt* = 0.2, typical simulations can be completed within a few tens of iterations (*i* form 1 to *N*_T_, *N*_T_ = 40 for the adaptation task, 60 for the controlled oscillation, and 30 for the gap gene example). We use the forward Euler discretization mainly for simplicity, and are fully aware that a large timestep may introduce large numerical error. Yet, the aim of employing NN in this paper is to search for necessary logical regulatory connections (i.e., network topologies), which are actually qualitative features. If in some future cases numerical accuracy do matters a lot, the neural-ODE framework^19^ can be employed to train the RNN with much higher numerical accuracy.

The initial condition should also be specified to complete the definition of a dynamic system. For Figs. 1–4, initial value of the output node *g*_1_ is set to be the desired output at the pre-stimulus state (0.4 for the adaptation task, and 0.1 for the controlled oscillation task). Initial pre-stimulus value of the other nodes (${g}_{2}$, etc.) are set to be trainable variables. For the gap gene case, the initial condition for all gap genes are simply zero everywhere, representing a newly formed fertilized egg before the expression of zygotic genes. In the CA example, initial values of all genes are provided explicitly by the first frame of the time-course of training data. All detailed information concerning model definition and training data selection is listed in Table S1.

### The link-mutation technique

The trained NN represents a black-box function *F* with input layer $\left({g}_{1},\cdots ,{g}_{m}\right)$ and output layer $\left({f}_{1},\cdots ,{f}_{n}\right)$. Perturbing the regulation link from *g*_1_ to *g*_2_ can be implemented by running the function *F* twice with different inputs: (where 0 < *λ* < 1)

5 $$\begin{array}{c}\left({f}_{1},{f}_{2},{f}_{3},\cdots ,{f}_{n}\right)=F\left({g}_{1},{g}_{2},\cdots ,{g}_{m}\right)\\ \left({f}_{1}^{{\prime} },{f}_{2}^{{\prime} },{f}_{3}^{{\prime} },\cdots ,{f}_{n}^{{\prime} }\right)=F\left(\lambda {g}_{1},{g}_{2},\cdots ,{g}_{m}\right)\end{array}$$

And stacking the output dimensions $\left({f}_{1},{f}_{2}^{{\prime} },{f}_{3},\cdots ,{f}_{n}\right)$ gives the perturbed ***f*** term. That is, replace ${g}_{1}$ value with the discounted value $\lambda {g}_{1}$ when and only when computing ${f}_{2}$. This kind of sensitivity analysis procedure can be applied to arbitrary black-box function. Comparison of different detailed implementations of the link mutation method is listed in Table S2.

### Enumeration with Hill-function model

Enumerating all topologies is straightforward. The directed link between each pair of nodes (8 links in total) are allowed to be activating, inhibiting, or non-exist. Multiple regulations, like ${g}_{1}$ is at the same time activated and inhibited by ${g}_{2}$, are not allowed. Among these 3^8^ topologies, 2304 satisfy basic requirements of connectivity. That is, both inputs should be connected to the core network, and ${g}_{1}$ and ${g}_{2}$ should be connected bidirectionally to keep the 2-node network irreducible to a single-node one. These 2304 topologies, having 4 to 8 links, are used for subsequent searching.

Hill function model of regulation links are implemented as follows. Activation and repression are formulated by terms ${h}^{+}$ and ${h}^{-}$ respectively. For example, if ${g}_{i}$ is activated by ${g}_{j}$, and repressed by ${g}_{l}$, the corresponding HF terms are:

6 $${h}_{{ij}}^{+}=\frac{{b}_{{ij}}{{g}_{j}}^{n}}{{{K}_{{ij}}}^{n}+{{g}_{j}}^{n}};\,{h}_{{il}}^{-}=\frac{{{K}_{{il}}}^{n}}{{{K}_{{il}}}^{n}+{{g}_{l}}^{n}}.$$

We set the Hill coefficient *n* = 2 in the enumerative study for simplicity. The synthesis rate ${f}_{i}$ in Eq. 1 integrates multiple regulatory effects of this kind. We take the convention that treats the Hill activation terms to be additive, and those repression terms to be multiplicative. Basal expression is ignored.

7 $${f}_{i}=\left(\mathop{\sum} _{j}{h}_{{ij}}^{+}\right)\left(\mathop{\prod}\limits_{l}{h}_{{il}}^{-}\right)$$

With this formulation, each activating link (each ${h}^{+}$ term) has two parameters *K* and *b*, and a repressive link only has one parameter *K*.

For each network topology, the parameters (*K* and *b*) are sampled in independently from the exponential distribution $p\left(x\right)={e}^{-x}.$ For each network topology, 160,000 sets of random parameters are sampled. A topology is considered to be “successful” only if no less than 2 successful parameter sets were obtained. Dynamic trajectories are simulated with forward Euler method for 200 steps; and we judged whether the output level oscillates first by simply calculating its temporal variance over steps 101~200, followed by a manual check. As the exhaustive search serves only as a validation step in this paper, there surely could be some omission and false positive, but they should not affect our general conclusions.

(All numerical simulation and data analysis in this paper are performed using custom code^36^ programmed in Python 3.6.5, Tensorflow 1.8.0 and MATLAB 2017a.)

### Reporting summary

Further information on research design is available in the Nature Research Reporting Summary linked to this article.

## Supplementary information

Supplementary Information

Reporting Summary

## Data Availability

Minimal datasets for the generation of the main figures are deposited to GitHub at https://github.com/sjx93/rnn_for_gene_network_2020/tree/v1.0. The raw datasets generated and/or analyzed during the current study are available without any restrictions within a month from the corresponding author on reasonable request.

## Code availability

The custom code generated during the current study are available in the *Zenodo* repository, https://zenodo.org/record/4705184. DOI: 10.5281/zenodo.4705184^36^
