# Supplementary material for: Finding gene network topologies for given biological function with recurrent neural network
Source: Nat Commun. 2021 May 25;12:3125. doi: 10.1038/s41467-021-23420-5 (PMC8149884; doi:10.1038/s41467-021-23420-5)
Supplement: Supplementary file 1 — Supplementary Information [file 41467_2021_23420_MOESM1_ESM.pdf]

Supplemental Information for

Finding Gene Network Topologies for Given Biological Function with Recurrent Neural Network

Jingxiang Shen, Feng Liu, Yuhai Tu and Chao Tang

Correspondence to: tangc@pku.edu.cn

This PDF file includes:

Supplemental text S1 to S11

Figures S1 to S6

Table S1 to S5

Supplemental references

(S1) Derivative and the link-mutation method in revealing the learnt regulations

With the regulatory link from node i to j being knocked down by a factor λ, the NN output (synthesis term $\boldsymbol{f}$) changes accordingly. If this perturbed $\boldsymbol{f}$ function is integrated through time following equation 3, the resulting trajectory is called the "mutant trajectory" $\boldsymbol{g}^{(i,j)}$. And its difference with the unperturbed trajectory $\boldsymbol{g}^{WT}$ gives the "mutant trajectory measure" of the effective gene regulation.

$$W_{i\ to\ j} \equiv -\int_t \left(g_j^{(i,j)}(t) - g_j^{WT}(t)\right) dt \qquad \text{(S1)}$$

Another representation of the effective regulation is introduced in the main text as $\langle \Delta_{ij} \rangle_{WT}$, where $\Delta_{ij}(\boldsymbol{g}, \lambda)$ is defined by equation 2. To be specific,

$$\langle \Delta_{ij} \rangle_{WT} = \sum_{\{I(t)\}} \int_t f_j\left(I(t), \cdots, g_i^{WT}(t)\right) - f_j\left(I(t), \cdots, \lambda g_i^{WT}(t)\right) dt \qquad \text{(S2)}$$

where f here is the trained NN time evolution function, and $\boldsymbol{g}^{WT}(t)$ is the WT (unperturbed) response curve under stimuli $I(t)$. Note that this quantity is also averaged across several different strengths of the input signal (for the controlled oscillation task). For the final CA example, as there is no external input, we average Δ_{ij} over different random initial conditions.

A third way of defining a measure of the effective regulations of a learnt black box is to evaluate Δ_{ij} with respect to randomly sampled $\boldsymbol{g}$ values instead of the WT trajectory. This $\langle \Delta_{ij} \rangle_{random\ samples}$ is a much rougher estimation than $\langle \Delta_{ij} \rangle_{WT}$, since it ignores the fact that the NN function $\boldsymbol{f}$ is trained only around the WT trajectories.

Besides, the partial derivative $\partial f_j / \partial g_i$ also reflects the regulation effect of node i on node j. As discussed above, $\partial f_j / \partial g_i$ can also be evaluated with respect to randomly sampled $\boldsymbol{g}$ values or the WT trajectory $\boldsymbol{g}^{WT}$.

We compare all these measures using the 200 repeated training carried out for the controlled oscillation task (Fig. 3d-f). The results are shown in Table S2. There, "Num."

means the number of distinct topologies belongs to the corresponding category; and "Prob." means the probability for the RNN model, after being trained and interpreted by the corresponding method, to give network topology of the corresponding category (in parallel to Fig. 3e). Some methods seem to be better than others. Especially, Δ_{ij} and $\partial f_j/\partial g_i$ evaluated on random samples seems to be the worst cases. The interpretation methods used in results of main figures are listed as part of Table S1.

(S2) Direct regularization cannot sparsen the regulation network effectively.

Training with weight decay makes the NN weights sparser. But sparsity of the NN weights does not mean sparsity in the effective regulation network that it represents, as parameters in RNN do not have explicit correspondence to links in the regulation network. To justify this, we applied L2 normalization to all the NN weights and bias, but did not block any of the regulatory links (as we did in Fig. 2c-d). The strength of L2 normalization term (rate of weight decay) is increased from zero to a very large value, so large that the network can no longer be trained (Fig. S2a). We found that network #17, where weight decay is extremely strong, has the same topology as network #1. Besides, all successful solutions (small training loss, also good in the view of peak response and adaptation error) have all the links, no matter how strong the L2 term is.

Another kind of regularization studied here is much more sophisticated. The $\boldsymbol{f}$ term with the regulatory link *i*-to-*j* being knocked *out* (i.e., λ=0) is computed at each train step, and a L1 regularization is introduced as follows.

$$L1 = \sum_{ij} \left| \langle \Delta_{ij} \rangle_{WT} \right| \tag{S3}$$

This term, when being added to the training loss, penalizes all "active" gene regulations. Theoretically, it may help to suppress all unnecessary links and leave only the minimal network just like LASSO regression. However, with our implementation, this term does not sparsify the resulting regulation networks very efficiently (Fig. S2b). These regularization techniques may be further investigated in future works.

(S3) The cutoff for defining RNN-discovered topologies in Fig. 3d

In Fig. 3, we use the sign of equation S1 as a representation of the activating / inhibiting nature of the link i to j. The magnitude of W is kind of reflection of the regulation strength, on which we may apply a cutoff. We have already presented an argument in the main text, that too low or too high cutoff value all lead to less topologies emerged. This situation is shown in Fig. S3c. By varying the cutoff value, the black line stands for the total number of distinct topologies given by 200 repeated trainings of the RNN model, and the lighter and darker red lines stand for the overlapping with Hill function topologies following Fig. 3d. The cutoff value used in the main text is that corresponds to the *global maxima* of the *black* line. (Vertical dashed line in Fig. S3c).

Also note that for the case studied here, 200 repeated training sessions (with random initial weights) seem to have uncovered most of the HF-compatible topologies. Another 200 training sessions hardly lead to any new HF-compatible topologies (Fig. S3d).

(S4) Monotonicity and transferability to Hill function models

We perform cross check using the HF models in order to demonstrate the consistency between the RNN solutions and the "biological realizable" regulations. As for possible "non-biological" NN solutions, we have mentioned that these solutions may rely on some highly non-monotonic regulatory links. Therefore, monotonicity of all regulatory links may be a way for describing "biological relevance" mathematically.

We define monotonicity in the following way. For a regulation link i-to-j, Δ_{ij} (equation 2) is evaluated at different time points and under different input stimuli, resulting in a WT-ensemble of Δ_{ij}. We mark different samples in this ensemble by the upper suffix n. Being averaged over n, the mean value gives $\langle \Delta_{ij} \rangle_{WT}$. However, this ensemble of $\Delta_{ij}^{(n)}$ may not always have uniform signs – the link i-to-j may appear to be activating under some circumstances, while inhibiting in some other situations. Non-

monotonicity of a link *ij* is defined as the sum of the minority:

$$min\left(\sum\nolimits_{n\left|\Delta_{ij}^{(n)}<0\right.}\left|\Delta_{ij}^{(n)}\right|,\sum\nolimits_{n\left|\Delta_{ij}^{(n)}>0\right.}\left|\Delta_{ij}^{(n)}\right|\right)$$

And the non-monotonicity of an RNN solution is the sum of non-monotonicity of all its regulation links. The statistics are shown in Fig. S3e. Most RNN solutions that give rise to HF-relevant networks have low non-monotonicity value (Fig. S3e upper, marked by the arrow head). And the distribution of non-monotonicity for HF-irrelevant RNN solutions peaks at a much larger value (Fig. S3e lower). Monotonicity correlates strongly with HF-relevance.

Based on these observations, a natural postulation is that penalizing this non-monotonicity term during training may help to find HF-relevant solutions with high probability. It is indeed the case, though the improvement does not seem to be very significant (Table S2, part II. 200 repeated trainings are studied here).

(S5) Increasing the variance of NN weights at initialization does not help the RNN to explore the entire network topology space

We have mentioned in the main text that RNN has bias towards different feasible underlying mechanisms (Fig. 3f). Gradient descent is inherently a history-dependent searching algorithm; training may be led to and trapped at just a few local minima. Initializing the model parameters in a wider range, thus covering the attracting basins of more local minima, seems to be a straightforward solution to this limitation. However, this seems not to be applicable to NN. In this paper, initialization of the NN weights follows identical-independent Gaussian distribution. Changing the variance of weights at initialization do affects the network topologies found subsequently (Fig. S3f-h). With increasing initial variance from 0.01 to 0.1 to 1.0, the number of “RNN-relevant” topologies on the “left branch” also increases from 0 to 5 to 6. However, NN training is severely affected by too large initial variance (Fig. S3h), resulting in a dramatic decrease in the probability of finding a HF-relevant topology by RNN.

(S6) Modifying training details changes the bias of RNN

In the "default" training settings, the pre-stimulus level of g_1 should be 0.1 (training case 0 in Fig. 3b), while the pre-stimulus level of g_2 is a free parameter. And the loss function is simply the square root sum of all three training cases (Fig. 3b). Repeated training with these settings would give the solutions marked red in Fig. 3d. In a modified training scenario, the pre-stimulus level of g_2 is set to a fixed value 0.9. For the initial phase of training (steps 1 to 2000) loss function contains only the term for oscillation (training case 1 in Fig. 3b), while the full loss function (containing training cases 0, 1, and 2) is used for training steps after 2000. With these settings, RNN can be pushed to explore the left-hand-side branch (bright green in Fig. 3f). The green nodes are the "HF-compatible" topologies (identical or differ by only one more / one less link, as for the red case) within a total of 200 repeats.

(S7) Validation with Enumeration using Logistic regression model

Logistic regression model is a kind of generalized linear model. Mathematically, it is equivalent to single-layer NN:

$$f_i = \sigma\left(\sum\nolimits_j W_{ij} g_j + b_i\right); \quad \sigma(x) = \frac{1}{1 + e^{-x}}$$

Compared with a multi-layer (or deep) NN, this model is obviously much simpler and more transparent. As discussed in the main text, we need to verify that network topologies proposed by deep NN don not rely on any specialized nonlinearity, and can thus be transferred to other modeling schemes. Apart from the Hill-equation-based modelling scheme discussed in the main text, we conduct the same validation with Logistic regression models. Moreover, models with the Logistic-regression-like structure (linear + saturation) have been introduced by many authors to model the transcription regulation process [1,2].

With the optimal cutoff for regulation strength, 57 different network topologies emerged in 200 repeated training (same result as Fig. 3d-f); 38 of them (38/57=0.67) also appears in exhaustive search with the Logistic regression model. (For each network

topology, 160,000 sets of W and b parameters are randomly sampled.) Again, only the "right branch" of the network topology space was explored by NN. Though Hill equation and Logistic regression are very different model frameworks, the cross-validation results presented here and in Fig. 3d-f are in general consistent.

(S8) A repeat of the training-and-deletion sequences of Fig. 4a-b.

Different training-and-deletion sequences could lead to different sparse regulation modules. Fig. S5a shows a parallel run with the same settings as Fig. 4a-b. Although solution #1 in both cases have identical topology, differences in the effective regulation strength lead to different link deletion. These two cases deviate from each other significantly at step #3 and later. The sparse solution here (#5 in Fig. S5a) has effectively two nodes: g_2 serves as "external input" to the oscillatory module. While the case presented in main text (#6 of Fig. 4b) is truly three-node – g_2 functioning as part of a feedback loop there. As expected, networks #5 in Fig. S5a can also be transferred successfully to Hill-function models (Fig. S5b). Parameters of this HF model is shown in Table S5.

An interpretation of it (#5 in Fig. S5a) could be like this. In the absence of both inputs, the oscillatory module (feedback loop between g_1 and g_3) is suppressed by g_2. This repression is released by input I_1, yielding oscillatory response. On the other hand, I_2 is able to activate g_1 but unable to remove the repression on the other half of the feedback loop, leading to sustained high g_1 level.

(S9) Hill function model for Fig. 4e and Fig. S5b

The Hill function model used here is slightly more complicated than that used for enumeration in Fig. 4 (Methods). Here, Hill coefficient is set to n=4, and a basal expression term (u) is included:

$$f_i = \left(\sum_j h_{ij}^{+}\right)\left(\prod_l h_{il}^{-}\right) + u_i$$

Parameters K, b, and u are sampled from the exponential distribution $p(x) \propto e^{-0.5*x}$.

The parameter set that gives rise to Fig. 4e and Fig. S5b are given in Table S3 and S*, respectively. Numerical integration is carried out using forward Euler method, with a sufficiently small timestep dt=0.01.

(S10) Controlled oscillation with frequency modulation

Training target now consists of five parts: low g_1 in the absent of both stimuli, low/high frequency oscillation under low/high levels of I_1, and low/high steady-state response to low/high levels of I_2. Low and high input levels are set to be 0.4 and 0.8, respectively. Again, we use the technique of Fig. 4 to find minimum regulation networks, i.e., by removing irrelevant links sequentially. Also, for simplicity of the input links, I_1 and I_2 can only act on two different network nodes respectively. For the case that I_1 acts on g_2 and I_2 on g_1, a meaningful topology found is shown in Fig. S4b insert. The corresponding RNN behaves like Fig. S4a after training, where oscillation frequency increases with I_1 level.

This topology consists of two feedback loops, a small one g_1-g_3-g_1 and a large one g_1-g_3-g_2-g_1. The way it works can be interpreted as follows. The level of g_1 drops after a pulse, so as g_3 which relies solely on g_1 for activation. Then g_2 starts to decay without the activation from g_3. Only when g_2 falls below certain threshold, can it release its suppression on g_1 thereby trigger the next pulse. Therefore, the faster g_2 falls the higher the oscillation frequency. As for input I_1, its repression helps to cancel out high base-level expression of g_2, making it to fall faster when activation from g_3 disappears, hence modulate the oscillation frequency. In this sense, frequency modulation relies mainly on the large feedback loop (g_1-g_3-g_2-g_1), especially the link g_3-to-g_2. Without this link, the large feedback loop is broken, while other modules (small feedback loop and the double-negative path from I_1 to g_1) remains unaffected. This interpretation is supported by “mutating” this link in the trained network. As expected, though oscillatory response to I_1 persists, the frequency modulation property is lost (Fig. S4c-d).

(S11) Training the RNN to simulate CA models in Fig. 6

We first describe the preparation of training data. For each ground truth CA model being studied, 16 different (random) initial conditions are used to generate 16 different spatial-temporal trajectories. As the initial condition, all "cells" except the one in the center have all its $\boldsymbol{g}$ components equal to 0, and the central cell has randomly sampled values (between 0 and 1) for all g_i. Each trajectory has 21 pixels (cells) in the spatial direction (one-dimensional array, periodic boundary condition), and a total of 320 successive timesteps. The 320 time points are then down sampled by 10-fold, in order to add difficulty for reverse engineering. Therefore, the training data is an array of size [trajectories=16, timepoints=32, spatial-points=21, genes=10]. For each gene, its "expression levels" are then normalized by its maximum value in these 16*32*21 different situations.

The training data is then cut into shorter trajectories, each with 5 (course grained) time points, corresponding to 41 original time steps. The RNN model is initialized (t=0) using the first frame of it, and runs freely to compute 40 successive time points following equation 1. The model outputs at step t=10, 20, 30, 40 are compared with the rest 4 temporal frames of training data, giving the loss function.

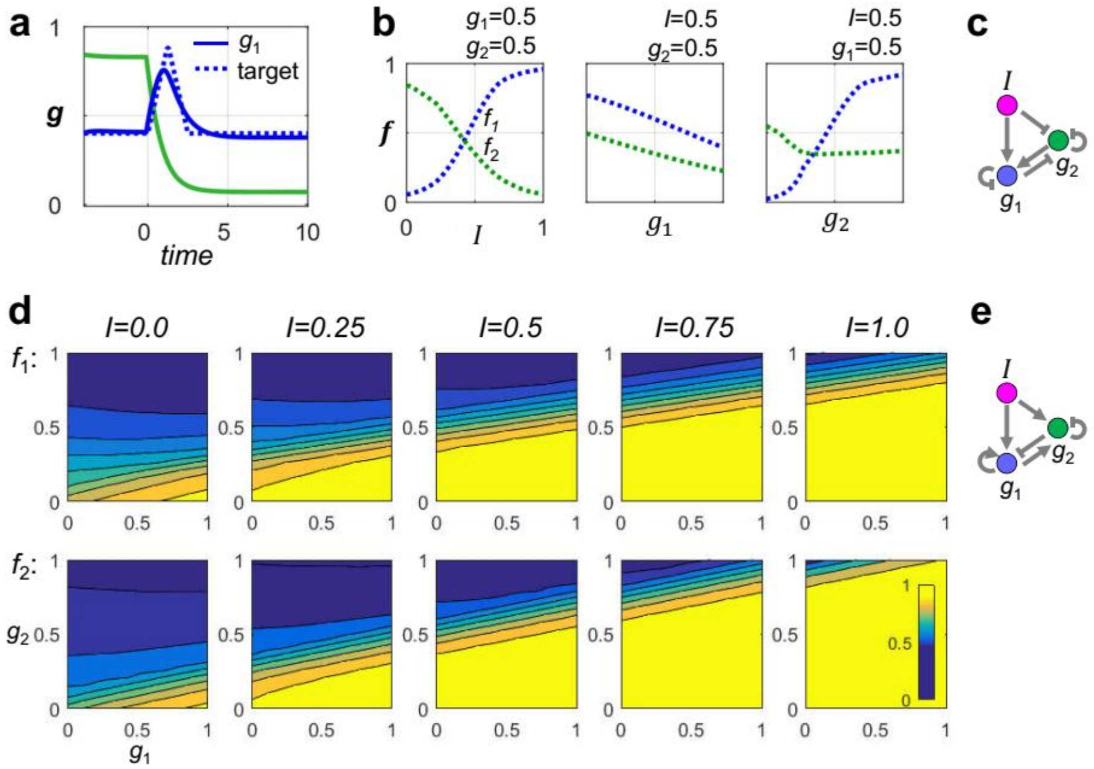

Fig. S1. Training the RNN to perform the adaptation task. (**a-c**) A triangular pulse instead of the smooth double-exponential curve were used as the target response curve. Training still converges, and the resulting RNN adopts a regulation network made up of the incoherent feed forward loop together with a feedback loop. (**d-e**) Direct visualization of the time iteration function fitted by NN. For the adaptation task, the NN computes the functions $f_1(g_1, g_2, I)$ and $f_2(g_1, g_2, I)$. In main text Fig. 1c, several cross sections of these multi-variable functions are plotted, reflecting the underlying regulations. Here, the entire function is shown, with the horizontal and vertical axis showing g_1 and g_2 levels, and different I level in different columns. Values of the synthesis term f are represented by color. The function is smooth and monotonic. The activating and inhibiting regulations indeed hold throughout the entire input range.

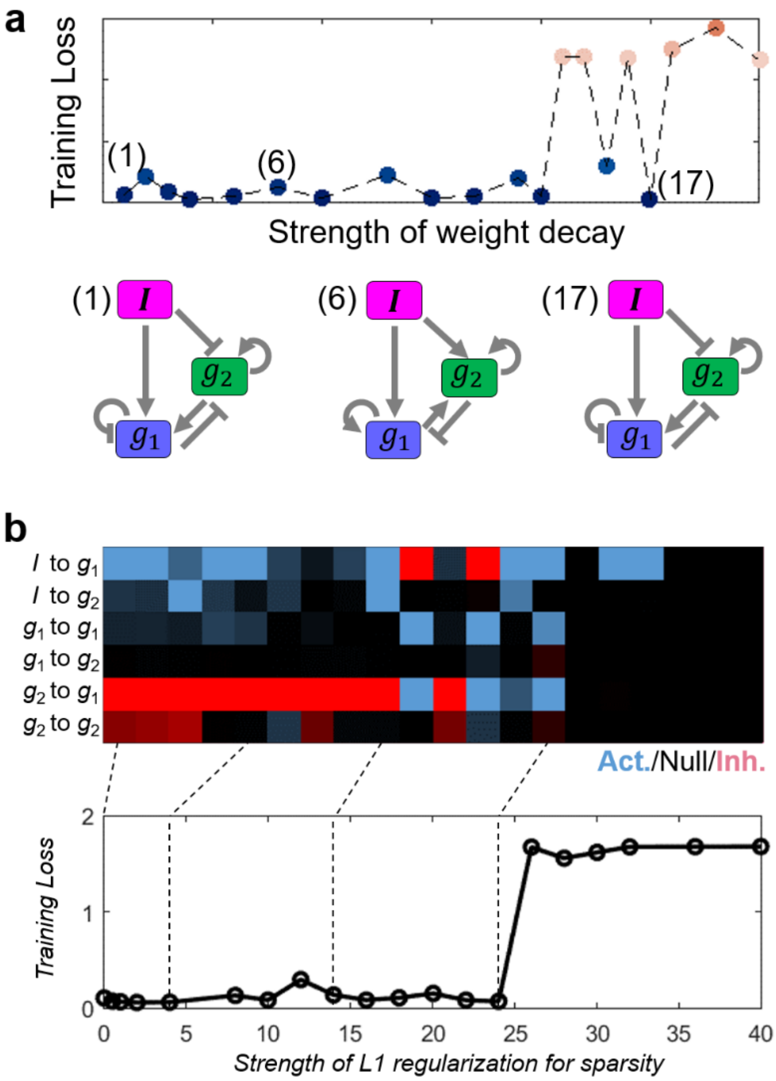

Fig. S2. Trying to find sparse regulation networks with regularization. (**a**) L2 regularization on NN weights and bias does not help sparsening the effective regulation network. Sparsity is not affected by increasing regularization strength. Successful solutions (ones with small training loss) have all six links, no matter how strong the L2 term is. (**b**) A different approach is to penalize $\sum_{ij}\left|\langle\Delta_{ij}\rangle_{WT}\right|$, i.e., the L1 norm describing the dependences between genes. However, sparsity seems not to be promoted by increasing the regularization strength, either.

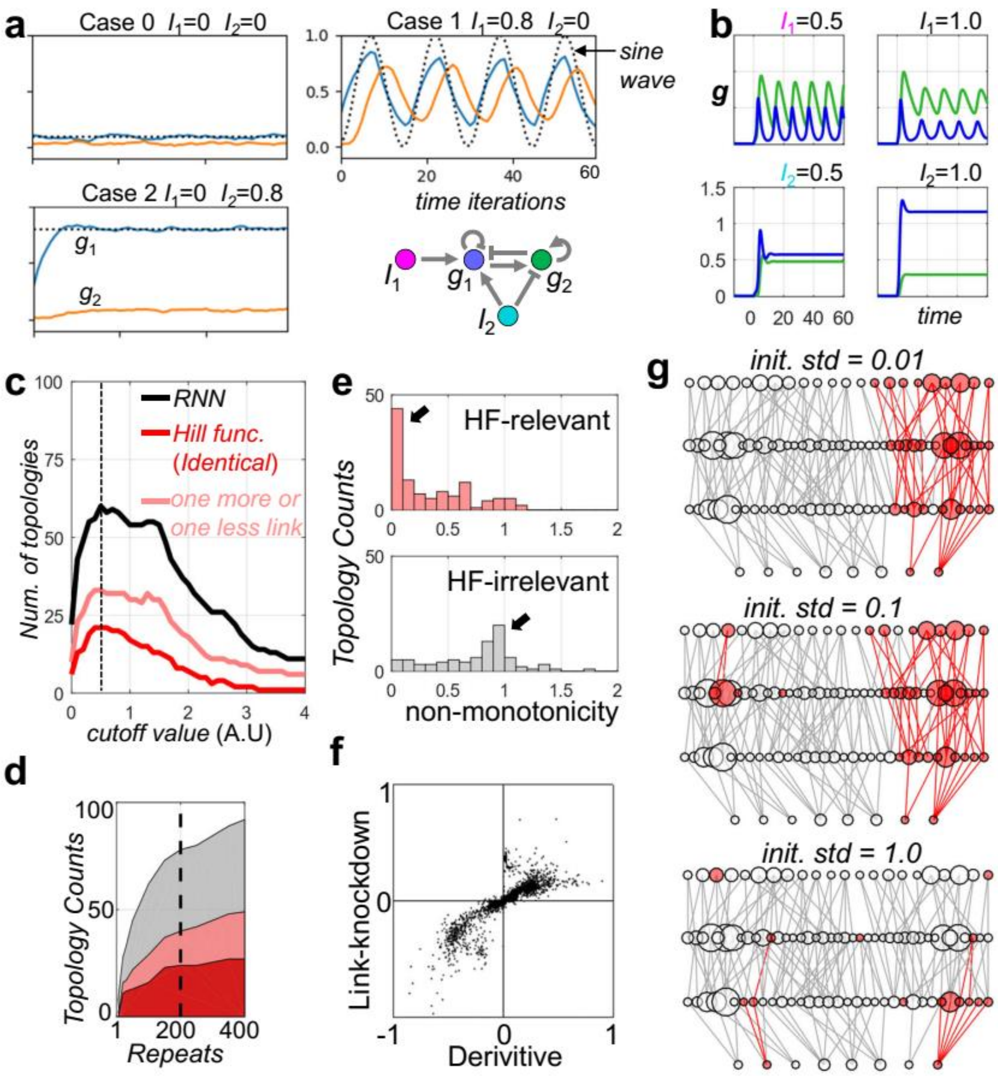

Fig. S3. The case of controlled oscillation. (**a**) Training of the RNN to perform this task does not rely on specific waveforms. Triangular wave is used in Figs. 3-4, and sine wave is used here. Both trainings are successful. (**b**) The network of Fig. 3c can be transferred to Hill-function models. Dynamic behavior of the HF model is shown. See Table S3 for the parameters. (**c**) The cutoff for defining topologies found by RNN. See Supplemental text S3 for details. (**d**) 200 repeated training sessions seem to have uncovered most of the HF-compatible topologies. (**e**) For a learnt regulation system, HF-relevance correlates with monotonicity. See Supplemental text S4 for details. (**f**) Derivatives $\partial f_j/\partial g_i$ also correlates positively with the regulations revealed by link mutation tests in the qualitative sense. (**g**) Compare this panel with Fig. 3f. Increasing the variance of NN weights at initialization do help the RNN to explore the left branch of solution, but training is severely affected by too large initial variance.

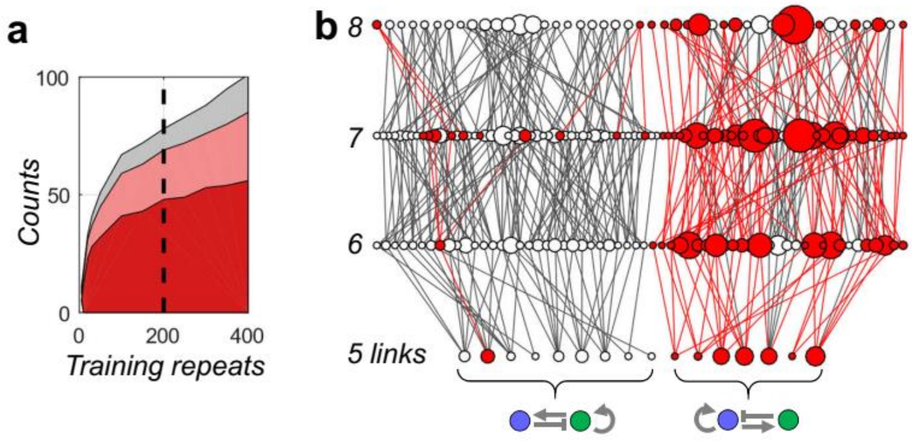

Fig. S4. Most topologies found by RNN can be transferred to Logistic regression model. (**a**) Statistics of the comparison between RNN with Logistic regression model based enumeration. Logistic regression model is equivalent to single-layered neural network. It is understandable that multi-layered NN bears more similarities with single-layered NN, than with Hill function models. (**b**) Similar to the Hill-equation results, those RNN-compatible topologies (colored red) have some inherent structural bias (not uniformly distributed).

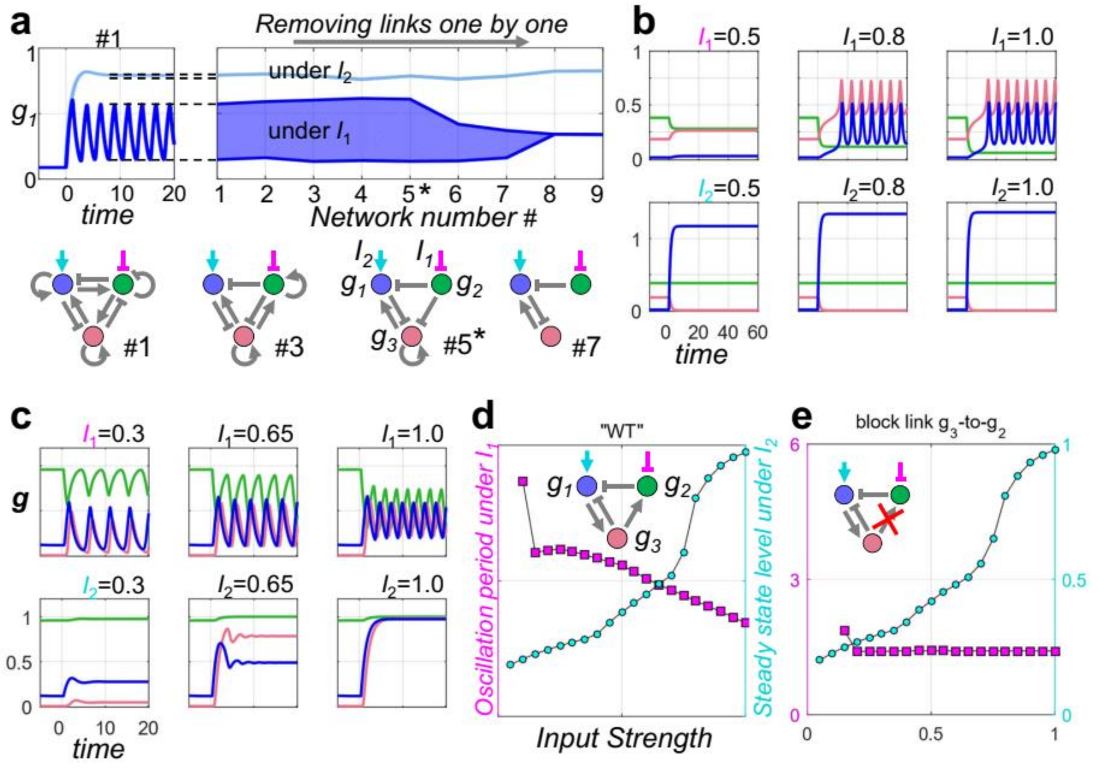

Fig. S5. (**a**) Another training-and-deletion sequence as Fig. 4a-b. Here, topology #5 is sparse and interpretable. It can also be successfully transferred to Hill function model (**b**). See Table S5 for the parameters. (**c**) Controlled oscillation with frequency modulation can also be achieved with the RNN based model. This panel shows the dynamics of the trained system. Oscillation frequency can be tuned by level of I_1, so as the steady-state response level by I_2. (**d**) The system in panel c shows clear dependence of oscillation period on the level of I_1 (magenta squares). Its effective network topology is shown as inset. See Supplemental text S3 for an interpretation. (**e**) Removing g_3-to-g_2 activation of the network in panel d destroys frequency modulation. Panels (d) and (e) have the same range of axis.

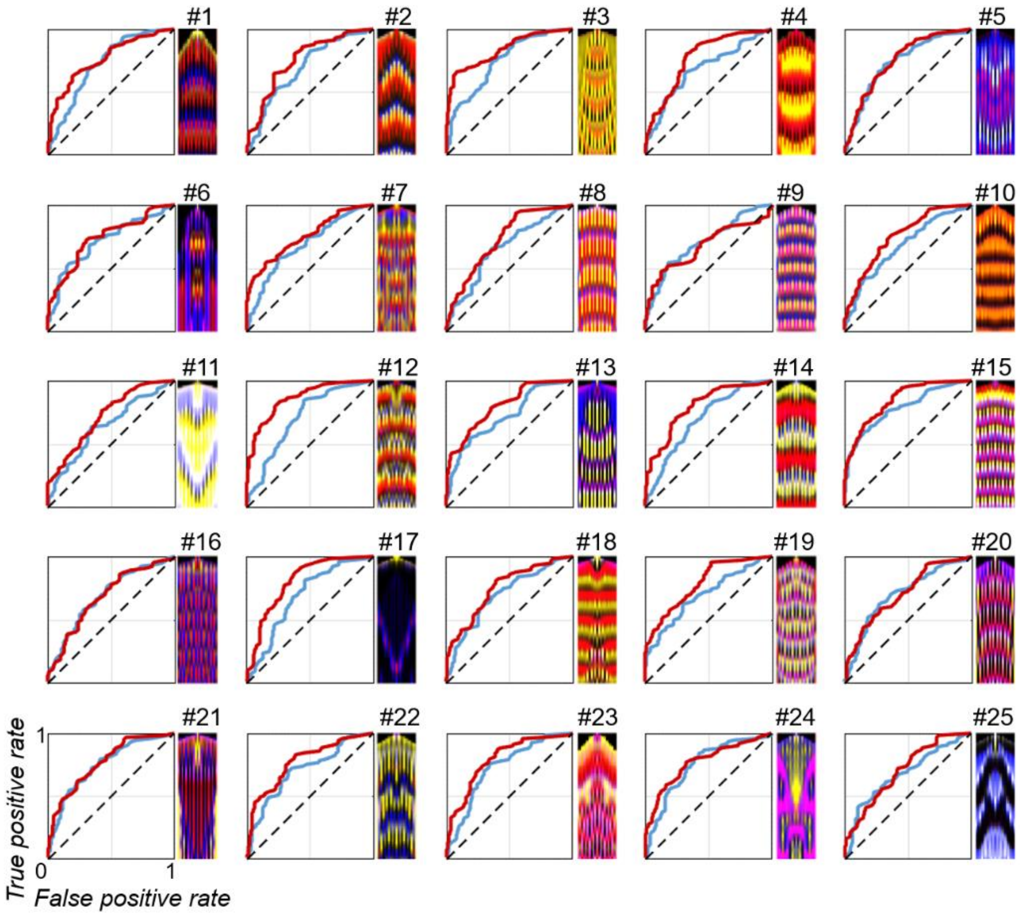

Fig. S6. All 25 CA models studied. Typical spatial-temporal dynamics of the ground-truth model is shown on the right. Although there are 10 genes in total (g_1- g_{10}), only g_1, g_2 and g_3 are shown in red, yellow and blue here. ROC curves for predicted activating /inhibiting links with our RNN-based method (similar to Fig. 6f) are shown on the left.

Table S1. RNN model settings for results in main text.

	Fig.1	Fig.2	Fig.3	Fig.4	Fig.5	Fig.6
Part I. RNN model structure						
Task	adaptation		Controlled oscillation		Gap gene	CA
Num. genes	2		2	3	4	10
Num. external inputs	1		2		2	0
Architecture	MLP	MLPx2	MLPx2	MLPx3	MLP	MLP
Num. nodes in each layer	3,16,16,2	3,16,16,1	4,16,16,1	5,16,16,1	6,16,16,4	20,64,64,10
Num. weights and bias	370	706	738	1155	452	6154
RNN iterations	40		60		30	40
Part II. Training data and the training process						
Observable genes	g_1 only		g_1 only		All	All
Num. frames per trajectory	40		60		1	32
Spatial points	1		1		64	21
Num. trajectories	3		3		1	16
Training steps	2000		4000		4000	4000
Time required for training	65s	81s	210s	220s	120s	430s
Sampling 1.6E5 parameter sets per topology	60s		100s	350s	120s	240,000s
Total num. of topologies	198		2304	~11000	~10^{11}	~10^{95}
Part III. Interpretation of the trained NN						
Method	plot	Mutant trajectory			$\langle \Delta_{ij} \rangle_{WT}$	
λ value	/	0	0.9	0.9	0	

Table S2. Comparison of different implementations of link-mutation method.

	All topologies	HF-relevant		Direct hit	
	Num.	Num.	Prob.	Num.	Prob.
Part I. For the 200 repeated trainings in Fig. 3d-f					
Mutant trajectory $\lambda = 0$	66	31	52.9%	15	28.0%
Mutant trajectory $\lambda = 0.5$	64	33		17	
Mutant trajectory $\lambda = 0.9$*	64	36	54.9%	21	38.3%
Mutant trajectory $\lambda = 0.95$	68	36		22	
Mutant trajectory $\lambda = 0.99$	65	40	58.0%	23	36.9%
WT trajectory $\lambda = 0$	52	25	52.6%	13	26.3%
WT trajectory $\lambda = 0.9$	64	39	68.1%	16	30.9%
WT trajectory $\lambda = 0.99$	61	40	66.7%	17	30.2%
Random samples $\lambda = 0$	52	27	57.0%	9	10.9%
Random samples $\lambda = 0.9$	55	33	68.2%	15	22.6%
Random samples $\lambda = 0.99$	64	40	67.7%	15	16.9%
WT trajectory $\partial f_j / \partial g_i$	63	34	63.9%	16	27.8%
Random samples $\partial f_j / \partial g_i$	55	30	46.4%	12	13.9%
Part II. Training with L1 regularization on monotonicity					
Mutant trajectory $\lambda = 0$	50	32	70.3%	18	42.2%
Mutant trajectoryλ= 0.9	52	30	65.9%	14	40.5%
Mutant trajectory $\lambda = 0.99$	51	26	57.3%	14	37.1%

Table S3. Parameters for Hill function model with the topology of Fig. 3c. (Hill coefficient n=2)

link	Activation/Inhibition	b	K
I_1 to g_1	Act.	0.174	0.523
I_2 to g_1	Act.	0.782	1.857
g_1 to g_1	Act.	1.689	0.339
g_2 to g_1	Inh.	/	0.420
I_1 to g_2	Act.	0.143	0.491
I_2 to g_2	Inh.	/	0.597
g_1 to g_2	Act.	1.403	0.753
g_2 to g_2	Act.	1.328	0.891

Table S4. Parameters for Hill function model in Fig. 4e. (Hill coefficient n=4)

link	Activation/Inhibition	b	K
I_1 to g_1	/	/	/
I_2 to g_1	Act.	0.983	0.397
g_1 to g_1	/	/	/
g_2 to g_1	Act.	1.259	0.730
g_3 to g_1	Inh.	/	4.503
I_1 to g_2	Act.	6.424	0.416
I_2 to g_2	/	/	/
g_1 to g_2	/	/	/
g_2 to g_2	/	/	/
g_3 to g_2	Inh.	/	0.043
I_1 to g_3	/	/	/
I_2 to g_3	/	/	/
g_1 to g_3	Act.	2.189	0.905
g_2 to g_3	/	/	/
g_3 to g_3	/	/	/

	Basal expression u
g_1	0.076
g_2	0.871
g_3	0.050

Table S5. Parameters for Hill function model in Fig. S5b. (Hill coefficient n=4)

link	Activation/Inhibition	b	K
I_1 to g_1	/	/	/
I_2 to g_1	Act.	1.370	0.324
g_1 to g_1	/	/	/
g_2 to g_1	Inh.	/	4.514
g_3 to g_1	Act.	2.960	0.381
I_1 to g_2	Inh.	/	0.643
I_2 to g_2	/	/	/
g_1 to g_2	/	/	/
g_2 to g_2	/	/	/
g_3 to g_2	/	/	/
I_1 to g_3	/	/	/
I_2 to g_3	/	/	/
g_1 to g_3	Inh.	/	0.446
g_2 to g_3	Inh.	/	0.440
g_3 to g_3	Act.	1.307	0.350
	Basal expression u		
g_1	0.0085		
g_2	0.382		
g_3	0.112		

References:

1. Jaeger, J., *et al.* Dynamic control of positional information in the early *Drosophila* embryo. *Nature* **430**, 368-371 (2004).
2. Cotterell, J. & Sharpe, J. An atlas of gene regulatory networks reveals multiple three-gene mechanisms for interpreting morphogen gradients. *Mol. Syst. Biol.* **6**, 425 (2010).
